# The Hyperbilirubinemia and Potential Predictors Influence on Long-Term Outcomes in Sepsis: A Population-Based Propensity Score-Matched Study

**DOI:** 10.3389/fmed.2021.713917

**Published:** 2021-09-17

**Authors:** Milin Peng, Fuxing Deng, Desheng Qi, Zhonghua Hu, Lina Zhang

**Affiliations:** ^1^Department of Critical Care Medicine, Xiangya Hospital, Central South University, Changsha, China; ^2^National Clinical Research Center for Geriatric Disorders, Xiangya Hospital, Central South University, Changsha, China; ^3^Department of Emergency, Xiangya Hospital, Central South University, Changsha, China; ^4^Hunan Key Laboratory of Molecular Precision Medicine, Xiangya Hospital, Institute of Molecular Precision Medicine, Central South University, Changsha, China

**Keywords:** sepsis, liver, risk factors, mortality, hyperbilirubinemia

## Abstract

**Objective:** Although hyperbilirubinemia has been associated with mortality in patients who are critically ill, yet no clinical studies dissect the effect of dynamic change of hyperbilirubinemia on long-term septic prognosis. The study aims to investigate the specific stages of hyperbilirubinemia and potential risk factors on long-term outcomes in patients with sepsis.

**Methods:** In this retrospective observational cohort study, patients with sepsis, without previous chronic liver diseases, were identified from the Medical Information Mart for the Intensive Care III MIMIC-III database. We used propensity scores (PS) to adjust the baseline differences in septic patients with hyperbilirubinemia or not. The multivariate Cox was employed to investigate the predictors that influence a clinical outcome in sepsis.

**Results:** Of 2,784 patients with sepsis, hyperbilirubinemia occurred in 544 patients (19.5%). After PS matching, a survival curve demonstrated that patients with sepsis with the new onset of total bilirubin (TBIL) levels more than or equal to 5 mg/dl survived at significantly lower rates than those with TBIL levels <5 mg/dl. Multivariate Cox hazard analysis showed that patients with TBIL at more than or equal to 5 mg/dl during sepsis exhibit 1.608 times (95% CI: 1.228–2.106) higher risk of 1-year mortality than those with TBIL levels <5 mg/dl. Also, age above 65 years old, preexisting malignancy, a respiratory rate above 30 beats/min at admission, serum parameters levels within 24-h admission, containing international normalized ratio (INR) above 1.5, platelet <50^*^10^∧^9/L, lactate above 4 mmol/L, and bicarbonate <22 or above 29 mmol/L are the independent risk factors for long-term mortality of patients with sepsis.

**Conclusions:** After PS matching, serum TBIL levels at more than or equal to 5 mg/dl during hospitality are associated with increased long-term mortality for patients with sepsis. This study may provide clinicians with some cutoff values for early intervention, which may improve the prognosis of patients with sepsis.

## Introduction

Sepsis is defined as a life-threatening acute organ dysfunction secondary to infection. A high incidence rate and high mortality of sepsis make it one of the leading causes of death as a global health priority (1, 2). Notably, sepsis-induced organ dysfunction is an important predictor for poor prognosis (3–5). The liver plays a central role in homeostasis, immune surveillance, inflammation, and bacterial clearance (6, 7). A large body of evidence has suggested that the liver is the main target of sepsis and decompensation of liver function can trigger overwhelming inflammation, immune response, and organ damage in sepsis (8, 9). However, despite extensively studied lung, kidney, and heart injury in the course of sepsis, the question of whether dysfunction of the liver is associated with mortality or a poor outcome in sepsis remains unresolved. Hepatic dysfunction and hyperbilirubinemia commonly occur in patients who are critically ill with an incidence rate of 40% and up to 20% in patients with bacterial infection (10).

Hyperbilirubinemia may result from bacterial products or as a consequence of the response of the host to infection. The etiology of hyperbilirubinemia in patients who are critically ill is multifactorial, probably cholestasis or sclerosing cholangitis caused by circulating endotoxins, inflammation, hypoxia hepatitis, lower liver perfusion and ischemia, genetic and metabolic variations, and so on (11–16). Hyperbilirubinemia has been shown to represent an important marker of mortality and poor outcomes for patients who are critically ill (17, 18). Patients with a preexisting liver deficit, like cirrhosis, have a worse outcome of sepsis than ones without liver dysfunction due to impaired immunity (19). The underlying mechanism may relate to the reversal of bilirubin transport from intrahepatic toward the circulation, which is beneficial for relieving the high-energy burden for hepatocytes and serves as a metabolic and inflammatory stress response as well (20). Hence, elevated serum bilirubin might indicate the impairment of energy consumption due to liver injury, and total serum bilirubin level has been widely recognized as a powerful maker for assessing hepatic function compared with other serum activities on laboratory tests.

Thus far, several studies have demonstrated the potential effect of liver dysfunction on short-term mortality of patients with sepsis by using different definitions of hepatic dysfunction (21–23). Whereas, it is still unclear of the long-term impact of dynamic change of hyperbilirubinemia in patients with sepsis. The appendant result derived from PROWESS-SHOCK (Prospective Recombinant Human Activated Protein C Worldwide Evaluation in Severe Sepsis and Septic Shock) trial showed that liver dysfunction is associated with 180-day mortality in patients with septic shock by using criteria of serum bilirubin ≥20 mmol/L (24). However, as hyperbilirubinemia is multifactorial, there is no large study dissecting the effect of liver dysfunction on long-term mortality of more than 180 days in patients with general sepsis. Therefore, we performed a large cohort study to analyze the dynamic change of hyperbilirubinemia and its influence on outcomes in patients with sepsis by using an openly available US-based critical care database named Medical Information Mart for Intensive Care (MIMIC)-III v 1.4, which includes 52,963 ICU admissions. Our results revealed a significant association between total bilirubin (TBIL) levels with long-term mortality in patients with sepsis.

## Methods

### Study Population

We conducted the cohort study according to the STrengthening the Reporting of OBservational studies in Epidemiology (STROBE) statement by using the MIMIC-III database, a large, integrated, de-identified, open-free, comprehensive clinical dataset, comprised of all the patients admitted to the ICUs Beth Israel Deaconess Medical Center in Boston, MA, from June 2001 to October 2012. For all of the data that are deidentified, patient consent or ethics approval will not be needed. Demographics records, laboratory results, radiology examinations, diagnosis, clinical treatment parameters, and dates of death were also concluded. The diagnosed diseases by the physician were according to the International Classification of Diseases, 9th revision (ICD-9) on patient discharge. Since the study was an analysis of a third-party anonymized publicly available database with preexisting institutional review board (IRB) approval, approval from our institution was exempted.

Sepsis was defined according to Sepsis-3 criteria: the suspected infection and Sequential Organ Failure Assessment (SOFA) score was of 2 points or more (25, 26). Hyperbilirubinemia was diagnosed in patients with the new onset of serum TBIL at more than or equal to 2 mg/dl during the hospitalization. The included criteria were as follows: age ≥18 years old; patients without previous chronic liver diseases according to the recorded ICD-9 codes, including liver cirrhosis; at ICU admission more than 24 h; missing data <50%. The excluded criteria were preexisting bilirubin at more than or equal to 2 mg/dl before admission, and previous chronic liver diseases like chronic hepatitis, acute-on-chronic liver failure, cirrhosis, liver cancer, hepatobiliary duct-related tumors, or acute liver conditions like drug or toxin-induced hepatitis. After being included, all the patients with sepsis were divided into a hyperbilirubinemia group (serum TBIL during hospitalization at more than or equal to 2 mg/dl) and a non-hyperbilirubinemia group (serum TBIL <2 mg/dl).

### Measures and Variable Definition

For the patients in the study, we retrieved demographic and admission information from the database during the first 24 h of ICU admission, including age, gender, ethnicity (White, Hispanic, Black, or other), weight, time of admission or discharge, the severity of illness parameters: the SOFA score (clarified into four different strata: 2–4, 5–9, more than or equal to 10 scores) and the Elixhauser comorbidity score, and vital signs: heart rate (clarified into two different strata: <100 beats/min, more than or equal to 100 beats/min), and respiratory rate (clarified into two different strata: <30 beats/min, more than or equal to 30 beats/min). In addition, we routinely collected laboratory parameters within the first 24 h of ICU admission, including maximum levels of white blood cell count (WBC); levels of hemoglobin (g/dl); hematocrit; platelet levels (stratified into five different scales: more than or equal to 250^*^10^∧^9/L, at 150–249^*^10^∧^9/L, at 100–149^*^10^∧^9/L, at 50–99^*^10^∧^9/L, and <50^*^10^∧^9/L); serum potassium/sodium/chloride levels; serum bicarbonate levels (stratified into three different scales: <22 mmol/L, at 22–29 mmol/L, and more than 29 mmol/L); serum blood urea nitrogen (BUN)/creatinine; serum lactate levels (stratified into four different scales: at 0–2 mmol/L, at 2.1–4 mmol/L, at 4.1–10 mmol/L, and more than 10 mmol/L); international normalized ratio (INR, stratified into two different scales: ≤1.5, more than 1.5); arterial blood gas with PH, partial pressure of oxygen (pO2), partial pressure of carbon dioxide (pCO2); alanine transaminase (ALT)/aspartate transaminase (AST). We also collected parameters in the course of sepsis: use of mechanical ventilation, use of vasopressor agents, and use of sedative drugs. By collecting in 24, 48, and 72 h, 7 days, and the day of discharge after ICU admission, serum levels of TBIL were classified into four different scales: at 0–1.9, 2–4.9, 5–10 mg/dl, and more than 10 mg/dl.

We also included preexisting medical comorbidities according to the recorded ICD-9 codes, including congestive heart failure (CHF), renal disease, atrial fibrillation (AFIB), coronary artery disease (CAD), chronic obstructive pulmonary disease (COPD), stroke, and malignant tumor. The Kidney Disease: Improving Global Outcomes (KDIGO) clinical practice guidelines were used to define acute kidney injury (AKI). We conducted the follow-up at day 30 (30 days), 90 days, 180 days, and 1 year from the database.

### Statistical Analysis

Statistical analysis was performed using SPSS 23 (SPSS, Inc., Chicago, IL). The parameters with missing data of more than 50% were excluded from our study. The mean-value imputation algorithm was selected to substitute missing values. Baseline characteristics and clinical parameters after ICU admission between the hyperbilirubinemia group and the control group were compared. All continuous variables were expressed as means (SD) or medians (interquartile range, IQR) by using either Student *t*-test or Mann–Whitney U test as appropriate. Categorical variables were compared by the chi-square test or Fisher's exact test. The survival curves for patients with sepsis were plotted using the Kaplan-Meier method, and differences between the curves were assessed using the log-rank test. To identify the association between the dynamic change and the levels of hyperbilirubinemia and a long-term outcome in sepsis, Cox regression analysis was used. Univariate Cox proportional hazards regression of clinical parameters was performed to identify potential predictors. Hazard ratios (HRs) were calculated with 95% CIs as an estimate of the risk associated with a particular variable. To determine independent predictors of the composite end points, variables in univariate Cox analysis with *p* < 0.1 were entered into multivariate Cox proportional hazards regression, and the predictors were performed with a likelihood ratio-forward selection.

To account for selection bias and potential confounding factors between groups in comparison of an outcome, we used the propensity score (PS) matching (1:1) to balances covariates for those who had hyperbilirubinemia and those who had not (532 pairs). A multivariable logistic regression model with confounding baseline characteristics was used to calculate the PS for each patient as the predicted probability of the hyperbilirubinemia group. The following variables were adjusted (Table 1): age; sex; race/ethnicity; preexisting medical conditions: CHF, AFIB, CAD, COPD, stroke, malignant tumor, and chronic renal disease; Elixhauser comorbidity score; respiratory rate; and biochemical parameters. One-to-one nearest-neighbor matching without replacement with a caliper width of.1 was conducted. We evaluated the balance test after matching, with no significant difference with chi-square (*X*^2^ = 16.468, *P* = 0.870), which demonstrated a good balance following PS matching between the hyperbilirubinemia and non-hyperbilirubinemia groups.

**Table 1:** Demographic characteristics between hyperbilirubinemia and non-hyperbilirubinemia groups before propensity score matching.

**Parameters**	**Full cohort**		***P***
	**Hyperbilirubinemia group**	**No-hyperbilirubinemia group**	
	**(*n* = 544)**	**(*n* = 2240)**	
Age, mean (SD), y	67.19 (27)	67.27 (26)	0.777
Male, *n* (%)	317 (58.3)	1200 (53.6)	0.049
Race, *n* (%)			
White	381 (70.0)	1569 (70.0)	0.518
Hispanic	15 (2.8)	67 (3.0)	0.888
Black	42 (7.7)	236 (10.5)	0.055
Other	106 (19.5)	368 (16.4)	0.098
Weight, mean (SD)	81.0 (25)	80.0 (25)	0.292
Preexisting medical conditions, *n* (%)			
CHF	134 (24.6)	579 (25.8)	0.584
AFIB	175 (32.2)	592 (26.4)	0.009
COPD	53 (9.7)	334 (14.9)	0.001
CAD	117 (21.5)	480 (21.4)	0.954
Stroke	19 (3.5)	194 (8.7)	<0.001
Malignancy	152 (27.9)	476 (21.3)	0.001
Renal disease	84 (15.4)	489 (21.8)	0.001
SOFA score at admission, *n* (%)			<0.001
Score at 2~4	88 (16.2)	982 (43.8)^*^	
Score at 5~9	274 (50.4)	1004 (44.8)^*^	
Score at ≥10	182 (33.5)	254 (11.3)^*^	
Elixhauser comorbidity index, mean (SD)	5.00 (10)	4.00 (9)	<0.001
Heart rate ≥ 100 (beats/min), *n* (%)	362 (66.5)	1414 (63.1)	0.149
Respiratory rate ≥ 30 (beats/min), *n* (%)	248 (45.6)	860 (38.4)	0.002
**Laboratory parameters within the first 24 h of ICU admission**			
Maximum WBC, mean (SD)	14.40 (9.68)	13.90 (9.60)	0.202
Maximum hemoglobin, mean (SD)	12.00 (2.88)	11.80 (3.10)	0.189
Maximum platelet levels (10^∧^9/L), *n* (%)			<0.001
Platelet≥ 250	170 (31.3)	1035 (46.2)^*^	
Platelet at 150~249	210 (38.6)	845 (37.7)	
Platelet at 100~149	98 (18.0)	232 (10.4)^*^	
Platelet at 50~99	47 (8.6)	93 (4.2)^*^	
Platelet at <50	19 (3.5)	35 (1.6)^*^	
Maximum potassium, mean (SD)	4.50 (1.2)	4.50 (1.0)	0.734
Maximum sodium, mean (SD)	140.00 (6.0)	141.00 (5.0)	0.051
Maximum bicarbonate levels, *n* (%)			<0.001
At <22	192 (35.3)	558 (24.9)^*^	
At 22–29	314 (57.7)	1398 (62.4)^*^	
At >29	38 (7.0)	284 (12.7)^*^	
Maximum chloride, mean (SD)	108.00 (8.0)	108.00 (8.0)	0.926
Maximum Bun, mean (SD)	29.00 (29.75)	26.00 (26.00)	0.060
Maximum lactate levels(mmol/L), *n* (%)			<0.001
Lactate at 0–2	190 (34.9)	1029 (45.9)^*^	
Lactate at 2.1–4	171 (31.4)	767 (34.2)	
Lactate at 4.1–10	149 (27.4)	392 (17.5)^*^	
Lactate at >10	34 (6.3)	52 (2.3)^*^	
Maximum creatinine, mean (SD)	1.40 (1.30)	1.30 (1.20)	0.010
Maximum hematocrit, mean (SD)	35.3 (7.85)	35.5 (8.60)	0.838
Maximum INR levels, *n* (%)			
At >1.5	288 (52.9)	651 (29.1)	<0.001
Maximum PH, mean (SD)	7.40 (0.07)	7.40 (0.07)	0.377
Maximum pO2, mean (SD)	214.0 (113)	214.0 (142)	0.921
Maximum pCO2, mean (SD)	47.0 (11)	47.0 (9)	0.030
Maximum ALT, mean (SD)	117.00 (292.50)	30.00 (52.00)	<0.001
Maximum AST, mean (SD)	155.00 (366.75)	41.00 (77.00)	<0.001
Maximum total bilirubin at 24 h, mean (SD)	3.30 (3.10)	0.50 (0.50)	<0.001
Maximum total bilirubin at 48 h, mean (SD)	3.50 (3.50)	0.60 (0.50)	<0.001

*Continuous variables are reported as mean (Standard deviation, SD), and Categorical variables are reported as count (% of column total)*.

*CHF congestive heart failure, AFIB atrial fibrillation, CAD coronary artery disease, COPD chronic obstructive pulmonary disease, SOFA Sequential Organ Failure Assessment, WBC white blood cell, INR International Normalized Ratio, BUN blood urea nitrogen, AST aspartate transaminase, ALT alanine transaminase, pCO_2_ partial pressure of carbon dioxide, pO_2_ partial pressure of oxygen*.

* *Represent significant difference with P < 0.05*.

After matching, survival curves were computed and plotted using the Kaplan-Meier method. Univariate and multivariate Cox proportional hazards regression models were constructed to explore the independent risk factors of influence on long-term outcomes in sepsis. A *P* < 0.05 (two-sided) was considered significant.

## Results

**Figure 1:**
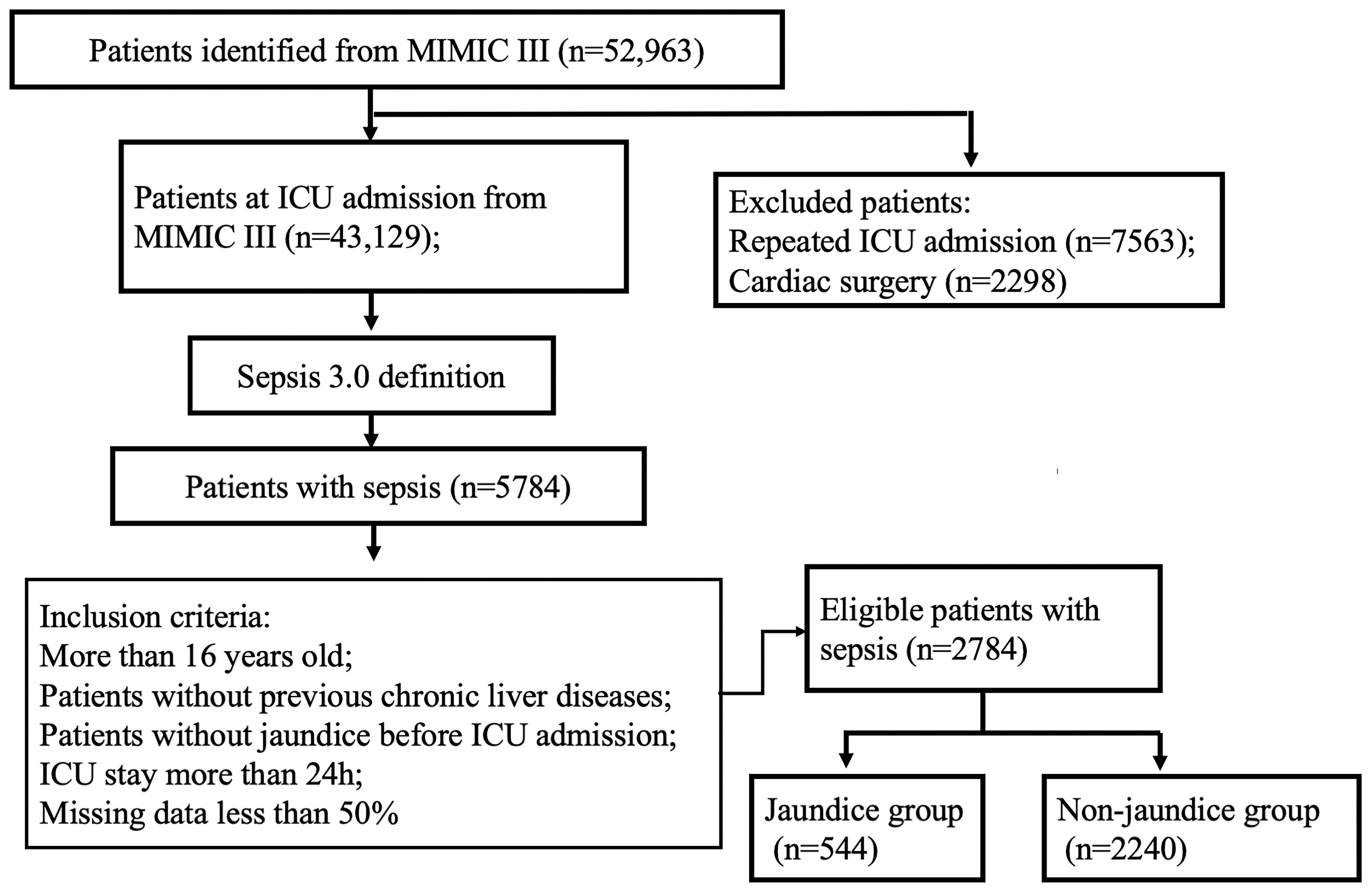
A flow chart of patient selection.

Of the 52,963 ICU admissions from the MIMIC-III database, 5,784 patients meet the definition of sepsis. About 3,310 participants were identified in our analysis according to the inclusion criteria; afterward, patients with preexisting liver disease (*n* = 526) were excluded from our study. Finally, 544 (19.5%) patients developed hyperbilirubinemia, while the remaining 2,240 (80.5%) patients did not (Figure 1).

### Patient Characteristics Before Matching

Table 1 shows the notable differences in baseline characteristics between the hyperbilirubinemia and non-hyperbilirubinemia groups of sepsis before PS matching. The results showed that men were prone to develop hyperbilirubinemia in the course of sepsis (58.3 vs. 53.6%; *P* = 0.049). The hyperbilirubinemia group had higher prevalence of preexisting medical comorbidities, including AFIB (32.2 vs. 26.4%; *P* = 0.009), and malignancy (27.9 vs. 21.3%; *P* = 0.001); lower prevalence of COPD (9.7 vs. 14.9%; *P* = 0.001), stroke (3.5 vs. 8.7%; *p* < 0.001), and renal disease (15.4 vs. 21.8%; *P* = 0.001) than the non-hyperbilirubinemia group (Table 1).

There was also a significantly higher severity of illness in the hyperbilirubinemia group than the non-hyperbilirubinemia group, with higher rates of the SOFA score at more than 4 (83.9 vs. 56.1%; *p* < 0.001), higher levels of Elixhauser comorbidity index [5. (10) vs. 4. (9)], and higher frequency of the respiratory rate at more than or equal to 30 beats/min (45.6 vs. 38.4%; *P* = 0.002; Table 1). At 24 h after admission to ICU, the hyperbilirubinemia group had significant higher rates of serum platelet levels at <150^*^10^∧^9/L (30.1 vs. 17.2%; *p* < 0.001), serum bicarbonate levels at <22 mmol/L (35.3 vs. 24.9%; *p* < 0.001), serum lactate levels at more than 4 mmol/L (33.7 vs. 29.8%; *p* < 0.001), and INR levels at more than 1.5 (52.9 vs. 29.1%; *p* < 0.001) when compared with the non-hyperbilirubinemia group (Table 1).

**Figure 2:**
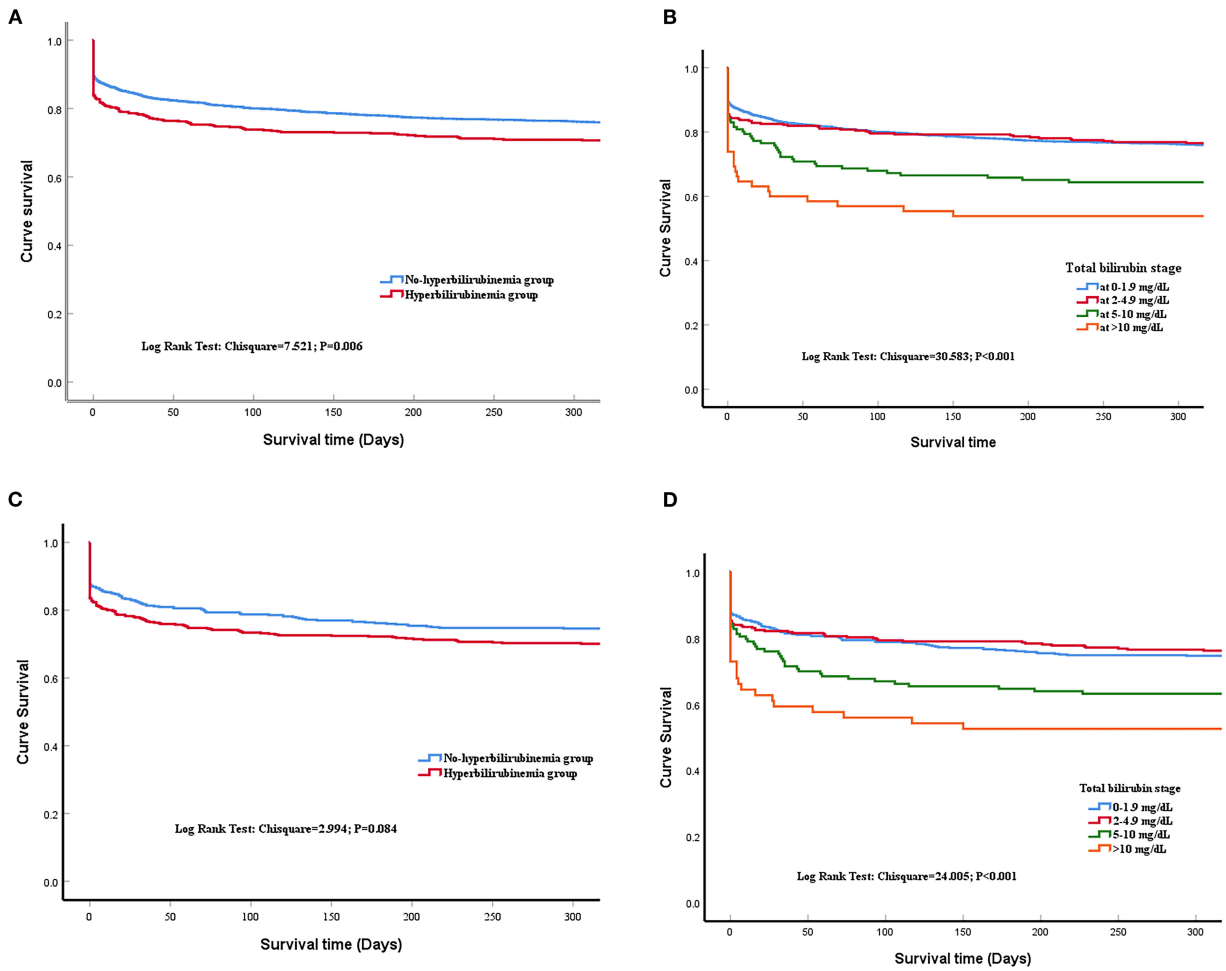
Time to death analysis. **(A)** shows the survival curves for all included patients at 1 year after admission of the last patients before matching, with a significant difference between liver injury and non-liver injury groups (Log-rank *P* = 0.006). **(B)** shows the survival curves for different stages of serum total bilirubin (TBIL) levels before propensity matching. Kaplan-Meier analysis showed survival time did not differ between the hyperbilirubinemia group and the normal group. Log-rank *p* < 0.001. **(C)** shows the survival curves between liver injury and non-liver injury groups after matching (log-rank *P* = 0.084); **(D)** shows the survival curves for different stages of serum TBIL levels after propensity matching (Log-rank *p* < 0.001).

The patients from the non-hyperbilirubinemia group all had TBIL levels below 1.9 mg/dl. By contrast, 62.1% of patients from the hyperbilirubinemia group had serum TBIL levels at 2–4.9 mg/dl, 25.9% had TBIL levels at 5–10 mg/dl, and 11.9% had TBIL levels at more than 10 mg/dl (Table 2). In addition, the hyperbilirubinemia group had higher rates of vasopressor usage when compared with the non-hyperbilirubinemia group. The overall in-hospital mortality of patients with sepsis was 23.5%. The patients in the hyperbilirubinemia group had significantly higher rates of in-hospital mortality (16.2 vs. 10.7%, *P* = 0.002), 30-day mortality (21.7 vs. 16.3%, *P* = 0.011), 90-day mortality (25.4 vs. 19.5%, *P* = 0.010), 180-day mortality (27.2 vs. 22.1%, *P* = 0.039), and 1-year mortality after discharge (29.8 vs. 24.5%, *P* = 0.037) than in the non-hyperbilirubinemia group, respectively (Table 2). Kaplan-Meier's analysis also showed that the 1-year survival rate was significantly lower in the hyperbilirubinemia group than in the non-hyperbilirubinemia group (*P* = 0.006) before matching (Figure 2A). Moreover, it is of note that TBIL levels more than or equal to 5 mg/dl at the hospital significantly increases the risk of mortality in sepsis (*p* < 0.001, Figure 2B).

**Table 2:** Clinical outcomes between hyperbilirubinemia and non-hyperbilirubinemia groups before propensity score matching.

**Parameters**	**Full cohort**		***P***
	**Hyperbilirubinemia group**	**No-hyperbilirubinemia group**	
	**(*n* = 544)**	**(*n* = 2,240)**	
Serum total bilirubin levels during the course of disease (mg/dL), *n* (%)			<0.001
Serum total bilirubin at 0–1.9 mg/dL	0 (0)	2240 (100.0)	
Serum total bilirubin at 2–4.9 mg/dL	338 (62.1)	0	
Serum total bilirubin at 5–10 mg/dL	141 (25.9)	0	
Serum total bilirubin at >10 mg/dL	65 (11.9)	0	
**The life support**			
Vasopressor usage, *n* (%)	251 (46.1)	878 (39.2)	0.003
Mechanical ventilation, *n* (%)	273 (50.2)	1208 (53.9)	0.125
Sedative drug usage	271 (49.8)	1143 (51.0)	0.633
AKI KDIGO stage, *n* (%)			0.097
AKI at 1 stage	118 (21.7)	532 (23.8)	
AKI at 2 stage	171 (31.4)	617 (27.5)	
AKI at 3 stage	101 (18.6)	370 (16.6)	
**Clinical outcomes**			
In-hospital mortality, *n* (%)	88 (16.2)	240 (10.7)	0.002
30 d mortality, *n* (%)	118 (21.7)	364 (16.3)	0.011
90 d mortality, *n* (%)	138 (25.4)	437 (19.5)	0.010
180 d mortality, *n* (%)	148 (27.2)	495 (22.1)	0.039
One-year mortality, *n* (%)	162 (29.8)	548 (24.5)	0.037

*Categorical variables are reported as count (% of column total)*.

*Abbreviations: AKI acute kidney injury, KDIGO the Kidney Disease: Improving Global Outcomes*.

### Univariate and Multivariate Cox Hazard Analysis of Risk Factors for Mortality in Sepsis Before Matching

Having identified that several laboratory serum activities, such as serum platelet levels, bicarbonate levels, lactate levels, and INR levels that are significantly associated with hyperbilirubinemia in ICU, we tested whether these serum activities, as well as other clinical outcomes, could be predictors of sepsis prognosis. We first deployed the univariate Cox proportional hazard model to analyze the predictors of mortality in sepsis. We employed a variety of categorical variables with a reference variable: serum platelet levels with platelet levels above or equal to 250^*^10^∧^9/L being a reference variable; serum lactate levels with lactate levels ≤ 2 mmol/L being a reference variable; serum bicarbonate levels with bicarbonate levels at 22–29 mmol/L being a reference variable; serum TBIL levels with TBIL levels <2 mg/dl being a reference variable; the AKI KDIGO stage without AKI as a reference variable.

Univariate analysis indicated that age at more than 65 years, weight, race in Black, Hispanic, or other, preexisting medical conditions (Malignancy, Stroke, AFIB, and renal disease), Elixhauser comorbidity index, respiratory rate at more than 30 m beats/min, serum INR levels at more than 1.5, serum platelet levels at <50^*^10^∧^9/L, serum lactate levels at more than 2 mmol/L, serum bicarbonate levels at more than 29 mmol/L, or <22 mmol/L, AKI at two or three stages, mechanical ventilation usage, vasoactive drug usage, and serum TBIL at more than or equal to 5 mg/dl were the significant risk factors in mortality in sepsis.

To test the prognostic predictors of mortality in sepsis, we performed multivariate analysis using the Cox proportional hazard model for all variables identified as significant by univariate analysis. Following control of confounders and Likelihood ratio (LR) forward elimination, results indicated (Table 3) that TBIL levels at 5–10 mg/dl during sepsis have a correlation with 1-year mortality risks [HR 1.396; 95% CI (1.040–1.875); *P* = 0.027]. The finding showed that age above 65 years old [Hazard ratio, HR 2.169; 95% CI (1.829–2.572); *p* < 0.001], other race [HR 1.391; 95% CI (1.158–1.671); *p* < 0.001], preexisting malignancy [HR 1.821; 95% CI (1.544–2.146); *p* < 0.001], preexisting stroke [HR 1.604; 95% CI (1.258–2.044); *p* < 0.001], Elixhauser comorbidity index at admission [HR 1.019; 95% CI (1.008–1.031); *P* = 0.001], respiratory rate above 30 beats/min at admission [HR 1.442; 95% CI (1.242–1.675); *p* < 0.001], serum INR above 1.5 at 24 h of admission [HR 1.217; 95% CI (1.038–1.426); *P* = 0.016], serum platelet <50^*^10^∧^9/L at 24 h of admission [HR 1.442; 95% CI (1.242–1.675); *p* < 0.001], serum lactate more than 10 mmol/L at 24 h of admission [HR 2.605; 95% CI (1.880–3.609); *p* < 0.001], serum bicarbonate <22 mmol/L [HR 1.375; 95% CI (1.162–1.626); *p* < 0.001], or above 29 mmol/L [HR 1.434; 95% CI (1.140–1.804); *P* = 0.002], and mechanical ventilation [HR 1.281; 95% CI (1.093–1.501); *P* = 0.002] were independent risk factors in 1-year mortality before matching by stepwise multivariate Cox hazard analysis.

**Table 3:** Univariate and stepwise multivariate Cox hazard analysis of risk factors for mortality in sepsis before matching.

**Parameters**	**Univariate analysis**			**Multivariate analysis**		
	**HR**	**95%CI**	***P***	**HR**	**95%CI**	***P***
Age>65 years	2.281	1.934–2.692	<0.001	2.169	1.829–2.572	<0.001
Weight	0.993	0.989–0.996	<0.001			
Gender (male)	0.924	0.798–1.071	0.294			
**Race**						
Race white	0.929	0.793–1.089	0.364			
Race black	0.760	0.580–0.997	0.047			
Race Hispanic	0.440	0.236–0.822	0.010	0.536	0.286–1.004	0.051
Race other	1.440	1.205–1.722	<0.001	1.391	1.158–1.671	<0.001
**Preexisting medical conditions**						
Malignancy	2.141	1.837–2.495	<0.001	1.821	1.544–2.146	<0.001
Stroke	1.530	1.205–1.944	<0.001	1.604	1.258–2.044	<0.001
AFIB	1.520	1.303–1.773	<0.001	-		
CAD	1.047	0.901–1.281	0.427			
Renal disease	1.337	1.129–1.583	0.001			
Elixhauser comorbidity index				1.019	1.008–1.031	0.001
Respiratory rate (> 30 beats/min)	1.441	1.244–1.670	<0.001	1.442	1.242–1.675	<0.001
Heart rate (> 100 beats/min)	1.100	0.942–1.284	0.227	-		
**Laboratory parameters within the first 24 h of ICU admission**						
INR levels at >1.5	1.486	1.279–1.725	<0.001	1.217	1.038–1.426	0.016
Maximum platelet levels (10^∧^9/L)						
Platelet ≥ 250						
Platelet at 150~249	0.805	0.680–0.953	0.012	0.791	0.668–0.938	0.007
Platelet at 100~149	1.041	0.826–1.312	0.735	0.877	0.691–1.112	0.278
Platelet at 50~99	1.043	0.748–1.453	0.806	1.274	0.815–1.990	0.288
Platelet at <50	1.575	1.023–2.426	0.039	1.442	1.242–1.675	<0.001
Maximum lactate levels (mmol/L)						
Lactate at 0–2						
Lactate at 2.1–4	1.233	1.035–1.469	0.019	1.121	0.938–1.339	0.208
Lactate at 4.1–10	1.492	1.225–1.816	<0.001	1.219	0.991–1.500	0.061
Lactate at >10	3.616	2.663–4.910	<0.001	2.605	1.880–3.609	<0.001
Maximum Bicarbonate levels				-		
Bicarbonate levels at 22–29						
Bicarbonate levels at <22	1.610	1.369–1.892	0.001	1.375	1.162–1.626	<0.001
Bicarbonate levels at >29	1.365	1.008–1.714	0.007	1.434	1.140–1.804	0.002
AKI KDIGO stage						
AKI at 1 stage	1.113	0.892–1.388	0.342			
AKI at 2 stage	1.653	1.361–2.008	<0.001			
AKI at 3 stage	1.704	1.369–2.120	<0.001			
Mechanical ventilation	1.297	1.118–1.506	0.001	1.281	1.093–1.501	0.002
Sedative drug usage	1.099	0.948–1.273	0.210			
The use of vasoactive drug	1.475	1.273–1.709	<0.001			
Serum total bilirubin levels during the course of disease (mg/dL)			
Serum total bilirubin at 0–1.9 mg/dL						
Serum total bilirubin at 2–4.9 mg/dL	0.977	0.772–1.235	0.844	0.830	0.651–1.058	0.132
Serum total bilirubin at 5–10 mg/dL	1.616	1.216–2.147	0.001	1.396	1.040–1.875	0.027
Serum total bilirubin at >10 mg/dL	2.203	1.525–3.182	<0.001	1.287	0.877–1.888	0.197

*AFIB atrial fibrillation, INR International Normalized Ratio, BUN blood urea nitrogen, KDIGO the Kidney Disease: Improving Global Outcomes*.

### PS Analysis

From the above results, we found that the baseline clinical information significantly influenced the hyperbilirubinemia group and long-term clinical outcomes. Thus, we applied propensity-score matching to minimize confounding biases. One-to-one propensity-score matching yielded a cohort of 532 patients in the hyperbilirubinemia group and 532 in the control group. Baseline characteristics for patients with and without hyperbilirubinemia were well-balanced after matching, as shown in Table 4. Standardized biases for all variables were.05 or less. The SOFA score at admission was excluded as a matching variable for one of its evaluated parameters, including TBIL. In the hyperbilirubinemia group after matching, the rates of the SOFA score at admission above 4 was significantly higher in the hyperbilirubinemia group when compared with the non-hyperbilirubinemia group (83.4 vs. 59.8%; *p* < 0.001).

**Table 4:** Demographic characteristics and clinical outcomes for hyperbilirubinemia and non-hyperbilirubinemia groups after propensity score matching.

**Parameters**	**Full cohort**		***P***
	**Hyperbilirubinemia group**	**No-hyperbilirubinemia group**	
	**(*n* = 532)**	**(*n* = 532)**	
Age, mean (SD), y	67.35 (26.96)	66.99 (26.09)	0.545
Male, *n* (%)	308 (57.9)	320 (60.2)	0.493
**Race**			
White	376 (72.4)	376 (70.7)	0.587
Hispanic	15 (2.8)	15 (2.8)	1.000
Black	39 (7.3)	49 (9.2)	0.316
Other	102 (19.2)	83 (15.6)	0.145
Weight, mean (SD)	81.00 (25.63)	79.75 (23.72)	0.355
Preexisting medical conditions, *n* (%)			
CHF	133 (25.0)	135 (25.4)	0.944
AFIB	171 (32.1)	154 (28.9)	0.287
COPD	53 (10.0)	54 (110.2)	1.000
CAD	116 (21.8)	121 (22.7)	0.768
Stroke	19 (3.6)	15 (2.8)	0.602
Malignancy	141 (26.5)	141 (26.5)	1.000
Renal disease	84 (15.8)	84 (15.8)	1.000
SOFA score at admission, *n* (%)			<0.001
Score at 2~4	88 (16.5)	214 (40.2)^*^	
Score at 5~9	271 (50.9)	244 (45.9)	
Score at ≥10	173 (32.5)	74 (13.9)^*^	
Elixhauser comorbidity index, mean (SD)	5.00 (10)	5.00 (9)	0.895
Heart rate ≥ 100 (beats/min), *n* (%)	351 (66.0)	368 (69.2)	0.295
Respiratory rate ≥ 30 (beats/min), *n* (%)	240 (45.1)	255 (47.9)	0.390
**Laboratory parameters within the first 24 h of ICU admission**			
Maximum WBC, mean (SD)	14.40 (9.58)	13.45 (10.60)	0.100
Maximum hemoglobin, mean (SD)	12.00 (2.80)	11.70 (3.20)	0.089
Maximum platelet levels (10^∧^9/L), *n* (%)			0.356
Platelet≥ 250	170 (32.0)	182 (34.2)	
Platelet at 150~249	209 (39.3)	198 (37.2)	
Platelet at 100~149	94 (17.7)	77 (14.5)	
Platelet at 50~99	43 (8.1)	53 (10.0)	
Platelet at <50	16 (3.0)	22 (4.1)	
Maximum potassium, mean (SD)	4.50 (1.2)	4.50 (1.2)	0.399
Maximum sodium, mean (SD)	140.00 (6.0)	140.00 (5.0)	0.326
Maximum bicarbonate levels, *n* (%)			0.174
At <22	184 34.6)	156 (29.3)	
At 22–29	310 (58.3)	332 (62.4)	
At >29	38 (7.1)	44 (8.3)	
Maximum chloride, mean (SD)	108.00 (8.0)	108.00 (7.0)	0.601
Maximum Bun, mean (SD)	28.50 (29.00)	26.50 (31.00)	0.967
Maximum lactate levels(mmol/L), *n* (%)			0.407
Lactate at 0–2	190 (35.7)	199 (37.4)	
Lactate at 2.1–4	170 (32.0)	186 (35.0)	
Lactate at 4.1–10	143 (26.9)	123 (23.1)	
Lactate at >10	29 (5.5)	24 (4.5)	
Maximum creatinine, mean (SD)	1.40 (1.28)	1.30 (1.30)	0.324
Maximum hematocrit, mean (SD)	35.5 (7.65)	34.9 (9.58)	0.157
Maximum INR levels, *n* (%)			
At >1.5	276 (51.9)	270 (50.8)	0.759
Maximum PH, mean (SD)	7.40 (0.07)	7.40 (0.07)	0.796
Maximum pO2, mean (SD)	214.0 (111)	214.0 (144)	0.501
Maximum pCO2, mean (SD)	47.0 (10)	47.0 (9)	0.262
Maximum ALT, mean (SD)	115.00 (288.00)	33.50 (70.75)	<0.001
Maximum AST, mean (SD)	152.50 (354.50)	43.00 (120.50)	<0.001
Maximum total bilirubin at 24 h, mean (SD)	3.30 (3.10)	0.60 (0.60)	<0.001
Maximum total bilirubin at 48 h, mean (SD)	2.10 (3.20)	0.00 (0.50)	<0.001

*Continuous variables are reported as mean (Standard deviation, SD), and Categorical variables are reported as count (% of column total)*.

*CHF congestive heart failure, AFIB atrial fibrillation, CAD coronary artery disease, COPD chronic obstructive pulmonary disease, SOFA Sequential Organ Failure Assessment, WBC white blood cell, INR International Normalized Ratio, BUN blood urea nitrogen, AST aspartate transaminase, ALT alanine transaminase, pCO2 partial pressure of carbon dioxide, pO2 partial pressure of oxygen*.

* *Represent significant difference with P < 0.05*.

The matched results also showed that the patients with sepsis with the new onset of hyperbilirubinemia were associated with significantly increased risks of mortality at the hospital (16. vs. 11.8%, *P* = 0.036), 30 days (21.4 vs. 16.7%, *P* = 0.038), 90 days (25 vs. 19.5%, *P* = 0.027), and 1 year (29.5 vs. 24.4%, *P* = 0.047), but not statistically increased in 90 days, 180 days, and 1-year mortality in the overall population (Table 5). The duration of mechanical ventilation, vasoactive drug or sedative drug usage, and AKI stage did not significantly differ between the two groups.

**Table 5:** Clinical outcomes between hyperbilirubinemia and non-hyperbilirubinemia groups after propensity score matching.

**Parameters**	**Full cohort**		***P***
	**Hyperbilirubinemia group**	**No-hyperbilirubinemia group**	
	**(*n* = 532)**	**(*n* = 532)**	
Serum total bilirubin levels during the course of disease (mg/dL), *n* (%)		<0.001	
Serum total bilirubin at 0–1.9 mg/dL	0 (0)	532 (100.0)	
Serum total bilirubin at 2–4.9 mg/dL	333 (62.6)	0	
Serum total bilirubin at 5–10 mg/dL	139 (26.1)	0	
Serum total bilirubin at >10 mg/dL	60 (11.3)	0	
**The life support**			
Vasopressor usage, *n* (%)	242 (45.5)	222 (41.7)	0.240
Mechanical ventilation, *n* (%)	267 (50.2)	263 (49.4)	0.854
Sedative drug usage	265 (49.8)	248 (46.6)	0.326
AKI KDIGO stage, *n* (%)			0.110
AKI at 1 stage	117 (22.0)	176 (23.1)	
AKI at 2 stage	167 (31.4)	132 (24.8)	
AKI at 3 stage	95 (17.9)	101 (19.0)	
**Clinical outcomes**			
In-hospital mortality, *n* (%)	85 (16.0)	63 (11.8)	0.036
30 d mortality, *n* (%)	114 (21.4)	89 (16.7)	0.038
90 d mortality, *n* (%)	133 (25.0)	104 (19.5)	0.027
180 d mortality, *n* (%)	143 (26.9)	120 (22.6)	0.066
One-year mortality, *n* (%)	157 (29.5)	130 (24.4)	0.047

*Categorical variables are reported as count (% of column total)*.

*AKI acute kidney injury, KDIGO the Kidney Disease: Improving Global Outcomes*.

After matching, Kaplan-Meier's analysis showed that the patients with new onset hyperbilirubinemia had a lower long-term survival rate compared with the non-hyperbilirubinemia group but did not reach statistical significance (*P* = 0.084; Figure 2C). However, TBIL levels more than or equal to 5 mg/dl at the hospital also significantly increases the risk of mortality in sepsis (*p* < 0.001, Figure 2D).

To further verify the levels of TBIL at the hospital on clinical outcomes, we then classified the patients into TBIL levels at the <5 mg/dl group and TBIL levels at the ≥5 mg/dl group. The results demonstrated that patients with sepsis with serum TBIL levels at ≥5 mg/dl during the hospital stage could bring significant poor outcomes on 30-day (28.1 vs. 17%), 180-day (34.7 vs. 19.4%), and 1-year mortality (37.7 vs. 21.7%), but with noninfluence on hospital mortality (18.6 vs. 12.8%) when compared with those with TBIL levels at the <5 mg/dl group (Supplementary Table 1).

### Univariate and Multivariate Cox Hazard Proportional Analysis to Explore Independent Predictors Affecting Long-Term Mortality in Sepsis After Matching

We then explored the direct effects of different levels of hyperbilirubinemia during the cause of sepsis on a long-term clinical outcome after matching. Multivariate analysis using the Cox proportional hazard model for all variables was identified as significant by univariate analysis. Following control of confounders and LR forward elimination, it was still shown in Table 6 that, when TBIL levels were <5 mg/dl as a reference variable, TBIL at ≥5 mg/dl during sepsis increased the risk of 1-year mortality with 1.608 times [95% CI (1.228–2.106); *P* = 0.001].

**Table 6:** Univariate and multivariate regression analysis to explore independent predictors affecting long-term mortality in sepsis after propensity score matching.

**Parameters**	**Univariate analysis**			**Multivariate analysis**		
	**HR**	**95%CI**	***P***	**HR**	**95%CI**	***P***
Age>65 years	2.041	1.583–2.633	<0.001	2.216	1.710–2.874	<0.001
Weight	0.998	0.993–1.003	0.369			
Gender (male)	0.890	0.705–1.124	0.329			
**Race**						
Race white	0.837	0.653–1.074	0.161			
Race black	0.706	0.432–1.152	0.164			
Race hispanic	0.330	0.106–1.029	0.056			
Race other	1.712	1.309–2.238	<0.001	1.647	1.256–2.160	<0.001
**Preexisting medical conditions**						
Malignancy	1.859	1.465–2.358	<0.001	1.688	1.314–2.167	<0.001
Stroke	1.282	0.702–2.342	0.419			
AFIB	1.550	1.223–1.965	<0.001	-		
CAD	1.178	0.904–1.537	0.226			
COPD	1.228	0.858–1.756	0.261			
Renal disease	1.354	1.013–1.809	0.041			
Elixhauser comorbidity index	1.047	1.030–1.064	<0.001			
Respiratory rate (>30 beats/min)	1.340	1.062–1.689	0.013	1.298	1.025–1.644	0.030
Heart rate (>100 beats/min)	1.184	0.919–1.527	0.192	-		
**Laboratory parameters within the first 24 h of ICU admission**						
INR levels at >1.5	1.690	1.331–2.147	<0.001	1.325	1.031–1.704	0.028
Maximum platelet levels (10^∧^9/L)						
Platelet≥ 250						
Platelet at 150~249	0.741	0.561–0.980	0.035	0.669	0.504–0.887	0.005
Platelet at 100~149	0.943	0.670–1.327	0.736	0.777	0.550–1.097	0.777
Platelet at 50~99	0.822	0.528–1.281	0.387	0.789	0.505–1.233	0.789
Platelet at <50	1.593	0.954–2.659	0.075	1.787	1.047–3.048	0.033
Maximum lactate levels(mmol/L)						
Lactate at 0–2						
Lactate at 2.1–4	1.227	0.905–1.663	0.189	1.107	0.813–1.507	0.518
Lactate at 4.1–10	1.715	1.264–2.328	0.001	1.435	1.042–1.977	0.027
Lactate at >10	4.990	3.354–7.423	<0.001	3.976	2.586–6.112	<0.001
Maximum Bicarbonate levels				-		
Bicarbonate levels at 22–29						
Bicarbonate levels at <22	1.982	1.555–2.526	<0.001	1.536	1.192–1.980	0.001
Bicarbonate levels at >29	1.487	0.970–2.279	0.068	1.767	1.147–2.725	0.010
AKI KDIGO stage						
AKI at 1 stage	0.921	0.638–1.328	0.659			
AKI at 2 stage	1.600	1.177–2.174	0.003			
AKI at 3 stage	1.868	1.346–2.593	<0.001			
Mechanical ventilation	1.459	1.155–1.845	0.002			
Sedative drug usage	1.293	1.025–1.631	0.030			
The use of vasoactive drug	1.447	1.148–1.824	0.002			
**Serum total bilirubin levels during the course of disease (mg/dL)**						
Serum total bilirubin < 5 mg/dL						
Serum total bilirubin at ≥ 5 mg/dL	1.776	1.370–2.301	<0.001	1.608	1.228–2.106	0.001

*CHF congestive heart failure, AFIB atrial fibrillation, CAD coronary artery disease, COPD chronic obstructive pulmonary disease, SOFA Sequential Organ Failure Assessment. AKI acute kidney injury, KDIGO the Kidney Disease: Improving Global Outcomes*.

We also found that age above 65 years old [HR 2.216; 95% CI (1.710–2.874); *p* < 0.001], other race [HR 1.647; 95% CI (1.256–2.160); *p* < 0.001], preexisting malignancy [HR 1.688; 95% CI (1.314–2.167); *p* < 0.001], respiratory rate above 30 beats/min at admission [HR 1.298; 95% CI (1.025–1.644); *P* = 0.030], serum INR above 1.5 at 24 h of admission [HR 1.325; 95% CI (1.031–1.704); *P* = *0.0*28], serum platelet <50^*^10^∧^9/L at 24 h of admission [HR 1.787; 95% CI (1.047–3.048); *P* = 0.033], serum lactate more than 4 mmol/L [HR 1.435; 95% CI (1.042–1.977); *P* = 0.027], or even more than 10 mmol/L at 24 h of admission [HR 3.976; 95% CI (2.586–6.112); *p* < 0.001], and serum bicarbonate <22 mmol/L [HR 1.536; 95% CI (1.192–1.980); *P* = 0.001], or above 29 mmol/L [HR 1.767; 95% CI (1.147–2.725); *P* = 0.010] were independent risk factors in 1-year mortality in sepsis after matchings. When using serum platelet ≥ 250^*^10^∧^9/L as a reference variable, the result demonstrated that serum platelet at 150–249^*^10^∧^9/L was the only protective factor in long-term mortality in sepsis [HR 0.669; 95% CI (0.504–0.887; *P* = 0.005)].

## Discussion

In this large cohort study, we find the incidence rate of the new onset of hyperbilirubinemia during sepsis is 19.5%, which is considerably lower than the rate of hyperbilirubinemia in other patients who are critically ill (14, 27). The difference is mainly due to the hyperbilirubinemia in our study that is defined as the new onset excluding past liver diseases, while previous studies included patients with preexisting chronic liver conditions (22, 28). Our data show that, after PS matching, the new onset of hyperbilirubinemia during sepsis cannot significantly increase the risk of long-term mortality, but the patients with TBIL levels are more than or equal to 5 mg/dl do. Further multivariate Cox hazard analysis shows that age above 65 years old, other race, preexisting malignancy, respiratory rate above 30 beats/min at admission, serum parameters levels within 24-h admission containing INR above 1.5, platelet <50^*^10^∧^9/L, lactate above 4 mmol/L, bicarbonate <22 or above 29 mmol/L, serum TBIL during sepsis at more than or equal to 5 mg/dl are the independent risk factors in 1-year mortality of patients with sepsis.

Furthermore, the low level of platelet, the INR level more than 1.5, the respiratory rate more than or equal to 30 (beats/min), the higher creatinine level, the higher arterial pCO2, and the lower bicarbonate level in the hyperbilirubinemia group compared with the non-hyperbilirubinemia group indicate that there is a close correlation between hyperbilirubinemia and the deterioration of hematologic, coagulation, respiratory, urinary, and acid-base balance systems function in sepsis. In stepwise multivariate Cox hazard analysis, Elixhauser comorbidity index, the respiratory rate above 30 beats/min at admission, serum INR above 1.5, serum platelet <50^*^10^∧^9/L, serum lactate more than 4 mmol/L, serum bicarbonate <22 mmol/L, or above 29 mmol/L at 24 h of admission, and mechanical ventilation as independent risk factors in 1-year mortality of patients with sepsis hint that hematologic, coagulation, respiratory, and acid-base systems that are imbalance at the early stage of sepsis predict poor prognosis of long-term mortality of sepsis. In addition, when serum TBIL at more than or equal to 5 mg/dl as a predictive factor in 1-year mortality of patients with sepsis consolidates our speculation: the new onset of hyperbilirubinemia reach at some extent aggravates sepsis prognosis.

Intriguingly, we observe that the hyperbilirubinemia group has a significantly higher rate of preexisting AFIB and malignancy but a lower rate of past COPD, stroke, and renal disease than the non-hyperbilirubinemia group. The mechanism of the potential relationship between the new onset of hyperbilirubinemia and past medical history in sepsis is not clear and needs further study. Multivariate Cox analysis results suggest that preexisting diseases, such as malignancy, affect the long-term survival of patients with sepsis, which is consistent with recent findings (29). Given that great bias was frequently generated when analyzing the sole effect of hyperbilirubinemia on sepsis, we used PS matching analysis to balance baseline characteristics to minimize confounding bias. After matching, baseline variants are well adjusted to parallel, thus reducing confounding bias to the maximum extent. Importantly, the new onset of hyperbilirubinemia when TBIL levels achieve at more than or equal to 5 mg/dl is associated with significantly increased long-term mortality of patients with sepsis after matching. Compared with the previous study, which did not use PS matching to strictly control possible confounders (24), our study provides solid evidence and comprehensive dissection of the relationship between the dynamic change of hyperbilirubinemia and sepsis prognosis, and, at the same time, we seek a cutoff value of TBIL levels associated with a poor outcome in sepsis, which may guide for early intervention for ICU physicians. Our results are consistent with previous basic research, which shows that the liver plays an important role in endotoxin-induced acute lung injury (30). Moreover, Zhang et al. (31) have proposed a Cox regression model with time-varying covariates, which is useful for evaluating the dynamic change of hyperbilirubinemia on clinical outcomes and can be used in further analysis. The liver is the key detoxification organ and the critical site to clear invasive pathogens and alleviate inflammation reaction by exerting innate immune system function (6, 7). Meanwhile, liver dysfunction and associated hyperbilirubinemia lead to inflammation and immune response out of control and cascading organ damage in sepsis (8, 9).

Furthermore, we explore the independent risk factors affecting 1-year mortality of patients with sepsis after PS matching, and we find that old age, preexisting malignancy, respiratory rate above 30 beats/min at admission, serum parameters within 24-h admission with INR above 1.5, serum platelet <50^*^10^∧^9/L, serum lactate more than 4 mmol/L, and bicarbonate <22 or above 29 mmol/L are the independent risk factors in 1-year mortality of patients with sepsis. These parameters may provide early warning of the prognosis of patients with sepsis. Previous basic pieces of research show that sepsis-induced acute kidney and myocardial injury are age-dependent (32, 33), and a recent clinical study has shown that the odds for mortality of patients with sepsis in ICU increase with age (34), which are consistent with our results. In qSOFA definition, expert consensus shows the respiratory rate of 22/min or greater, which is the predictor for a poor outcome of patients with sepsis (25); our results further demonstrate the respiratory rate of 30/min or greater at admission is the independent risk factor in long-term mortality. Abundant studies have focused on the correlation between the serum lactate level and short-term mortality of patients with sepsis; the lactate level ≥2 mmol/L around is demonstrated having the predictive value (35, 36). However, the cutoff of the lactate level for predicting long-term mortality of sepsis remains unclear. Our results show lactate above 4 mmol/L within 24-h admission is the independent predictor for 1-year mortality of sepsis, and patients with lactate above 10 mmol/L will bring a poor prognosis in sepsis. Of note, recent studies have shown that platelets play a vital role in immunological surveillance against pathogens invaders and contribute to innate immune system function (37, 38). Wong et al. demonstrated that platelets collaborate with macrophages to fight against certain blood-borne infections. In addition, the absence of the platelet resulted in the platelet being unable to localize to the sites of infection, leading to rapid death of the Kupffer cells and endothelium, followed by more leakage of plasma out of blood vessels, and even host mortality (39). This may explain why patients with sepsis with lower-level platelets have poor outcomes in our study. Besides, we find that bipolar serum bicarbonate levels are another risk factor in the long-term mortality of patients with sepsis. Previous experimental studies indicate that endotoxemia significantly decreases bile acid-independent bile flow (BAIBF) and associated biliary ${\text{HCO}}_{3}^{-}$ output (40), thus reducing the serum bicarbonate level. As there are no studies that have reported the correlation between the bicarbonate level and sepsis mortality, our study first shows that bicarbonate <22 or above 29 mmol/L is an independent predictor for 1-year mortality of sepsis.

To our knowledge, this is the first and largest study so far to dissect the correlation between the dynamic change of hyperbilirubinemia and outcomes of sepsis. However, there are several limitations to our study. First, we used the database from a single academic medical center in the USA; therefore, some of the cases from almost 10 years ago, as diagnosis or treatment strategies at that time, would be inconsistent with current guidelines, which brings great bias. There is residual confounding by variables not collected into the MIMIC-III database. However, we include all the patients with sepsis according to the uniform standard of Sepsis 3.0 and apply PSs analysis and match the baseline characteristics of the patients to eliminate confounding factors and decrease bias to the maximum. Second, there are a few missing data that bring bias, yet we delete the data whose missing percentage is larger than 50% to decrease bias. The mean-value imputation algorithm is selected to substitute missing values. Third, the single-centered design restricts generalizability to apply our conclusion to other regions, while we use a large sample size and PS matching analysis to guarantee the quality of our study.

## Conclusion

In conclusion, patients with serum TBIL at more than or equal to 5mg/dl during sepsis decrease survival rates after PS matching. In addition, we conclude that age above 65 years old, preexisting malignancy, respiratory rate above 30 beats/min at admission, serum parameter levels within 24-h admission, containing INR above 1.5, platelet <50^*^10^∧^9/L, lactate above 4 mmol/L, and bicarbonate <22 or above 29 mmol/L are independent risk predictors for long-term mortality of sepsis. Our study provides solid evidence and will rekindle the awareness of the risk factors leading to poor prognosis in sepsis.

## Data Availability Statement

The original contributions presented in the study are included in the article/Supplementary Files, further inquiries can be directed to the corresponding authors.

## Ethics Statement

The Ethics approval statement was approved by the Institutional Review Boards of Beth Israel Deaconess Medical Center (Boston, MA), and all the data were under a deidentification process to protect individual privacy and waived the need for a patient consent statement. Written informed consent from the participants' legal guardian/next of kin was not required to participate in this study in accordance with the national legislation and the institutional requirements.

## Author Contributions

MP, DQ, LZ, and ZH contributed to the conception and design of the research. MP and FD contributed to the acquisition and analysis of the data. MP, ZH, and LZ contributed to the interpretation of the results. MP and DQ drafted the manuscript. DQ, ZH, and LZ revised the manuscript. All authors have agreed to be fully accountable for ensuring the accuracy of the work, and approved the final manuscript.

## Funding

This work was supported by Changsha Municipal Natural Science Foundation (grant number: kq2007078).

## Conflict of Interest

The authors declare that the research was conducted in the absence of any commercial or financial relationships that could be construed as a potential conflict of interest.

## Publisher's Note

All claims expressed in this article are solely those of the authors and do not necessarily represent those of their affiliated organizations, or those of the publisher, the editors and the reviewers. Any product that may be evaluated in this article, or claim that may be made by its manufacturer, is not guaranteed or endorsed by the publisher.

## Abbreviations

MIMIC: medical information mart for intensive care
STROBE: STrengthening the Reporting of OBservational studies in Epidemiology
ICD-9: International Classification of Diseases 9th revision
IRB: institutional review board
SOFA: sequential organ failure assessment
TBIL: total bilirubin
CHF: congestive heart failure
AFIB: atrial fibrillation
CAD: coronary artery disease
COPD: chronic obstructive pulmonary disease
ALT: alanine aminotransaminase
AST: aspartate aminotransferase
CI: confidence interval
HR: hazard ratio
OR: odds ratio
pCO_2_: partial pressure of CO_2_
INR: international normalized ratio
BAIBF: bile acid-independent bile flow
USA: United States.
