# Supplementary material for: The Hyperbilirubinemia and Potential Predictors Influence on Long-Term Outcomes in Sepsis: A Population-Based Propensity Score-Matched Study
Source: Front Med (Lausanne). 2021 Sep 17;8:713917. doi: 10.3389/fmed.2021.713917 (PMC8484885; doi:10.3389/fmed.2021.713917)
Supplement: Supplementary file 1 [file Table_1.docx]

Supplement Table 1.Clinical outcomes between serum total bilirubin at < 5 mg/dL group and serum total bilirubin at ≥ 5 mg/dL group after propensity score matching.

| Parameters | Full Cohort |  | *P* |  |
| --- | --- | --- | --- | --- |
|  | Serum total bilirubin at ≥ 5 mg/dL group  (n=199) | Serum total bilirubin at < 5 mg/dL group (n=865) |  |  |
| **The life support** |  |  |  |  |
| Vasopressor usage, n (%) | 242(45.5) | 222(41.7) | 0.240 |  |
| Mechanical ventilation, n (%) | 106(53.3) | 424(49.0) | 0.307 |  |
| Sedative drug usage | 106(53.3) | 407(47.1) | 0.116 |  |
| **Clinical outcomes** |  |  |  |  |
| In-hospital mortality, n (%) | 37(18.6) | 111 (12.8) | 0.101 |  |
| 30d mortality, n (%) | 56 (28.1) | 147 (17.0) | 0.001 |  |
| 90d mortality, n (%) | 69 (34.7) | 168 (19.4) | <0.001 |  |
| 180d mortality, n (%) | 75 (37.7) | 188 (21.7) | <0.001 |  |
| One-year mortality, n (%) | 79 (41.1) | 208 (25.1) | <0.001 |  |

Categorical variables are reported as count (% of column total).
